# The role of CPEB family proteins in the nervous system function in the norm and pathology

**DOI:** 10.1186/s13578-021-00577-6

**Published:** 2021-03-31

**Authors:** Eugene Kozlov, Yulii V. Shidlovskii, Rudolf Gilmutdinov, Paul Schedl, Mariya Zhukova

**Affiliations:** 1 Laboratory of Gene Expression Regulation in Development, Institute of Gene Biology, Russian Academy of Sciences, Moscow, Russia 119334; 2 Department of Biology and General Genetics, Sechenov First Moscow State Medical University (Sechenov University), Moscow, Russia 119992; 3 Department of Molecular Biology, Princeton University, Princeton, NJ 08544-1014 USA

**Keywords:** CPEB, Translation, Prion, Neurogenesis, Long-term memory

## Abstract

Posttranscriptional gene regulation includes mRNA transport, localization, translation, and regulation of mRNA stability. CPEB (cytoplasmic polyadenylation element binding) family proteins bind to specific sites within the 3′-untranslated region and mediate poly- and deadenylation of transcripts, activating or repressing protein synthesis. As part of ribonucleoprotein complexes, the CPEB proteins participate in mRNA transport and localization to different sub-cellular compartments. The CPEB proteins are evolutionarily conserved and have similar functions in vertebrates and invertebrates. In the nervous system, the CPEB proteins are involved in cell division, neural development, learning, and memory. Here we consider the functional features of these proteins in the nervous system of phylogenetically distant organisms: *Drosophila*, a well-studied model, and mammals. Disruption of the CPEB proteins functioning is associated with various pathologies, such as autism spectrum disorder and brain cancer. At the same time, CPEB gene regulation can provide for a recovery of the brain function in patients with fragile X syndrome and Huntington's disease, making the CPEB genes promising targets for gene therapy.

## Background

The functioning of the nervous system is based on the ability of neurons to perceive, transmit, and store information encoded in electrical and chemical signals. The molecular basis for this function in response to stimulation includes alterations in the intracellular distribution of proteins and RNAs and changes in the number and quality of membrane receptors in synapses. One of the most important mechanisms underlying changes in the intracellular architecture of both an individual neuron and entire brain networks is the activation of translation of localized mRNAs mediated by cytoplasmic polyadenylation. Critical regulatory elements that encode signals for transport, anchoring and translational regulation including signals for regulating cytoplasmic polyadenylation (cytoplasmic polyadenylation elements: CPE) are present in the 3′-untranslated region (3′UTR) of mRNA. CPE sequences are recognized by CPEB family proteins, which are widespread in the animal kingdom and show extensive homology among different organisms.

**Fig. 1:**
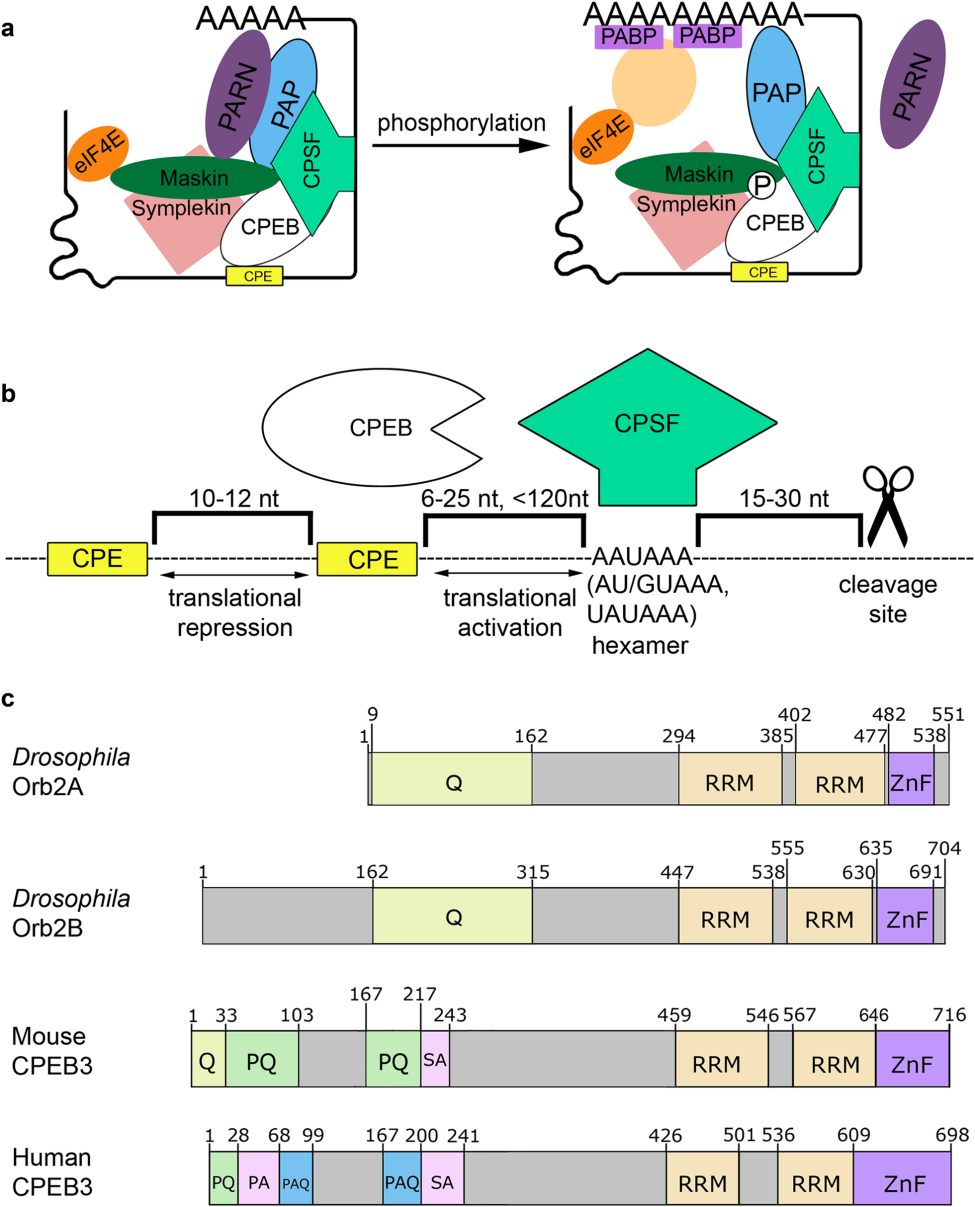
Properties of CPEBs and their interactions with other proteins in RNP complexes. **a** Simplified scheme of translational repression and activation that the CPEB protein exerts by regulating polyadenylation in *Xenopus*. Maskin interacts with CPEB and eIF4E, preventing the formation of the initiation complex. After CPEB phosphorylation, PARN dissociates from the complex and PAP initiates elongation of the poly(A) tail. PABP proteins bind to the poly(A) tail, promoting the formation of the initiation complex and dissociation of Maskin from eIF4E. **b** Optimal localization of binding sites in the 3’UTR of mRNA for translational regulation with the CPEB proteins in vertebrates. CPSF, cleavage and polyadenylation specificity factor. **c** Primary structure of the CPEB proteins with a prion-like domain at the N-terminus (drawn to scale). Numbers refer to amino acid positions. All CPEB proteins have RNA recognition motifs (RRMs) and a zinc finger domain (ZnF) at the C-terminus. Stretches of amino acids are colored: Q, glutamine rich; PQ, proline/glutamine rich; SA, serine/alanine rich; PA, proline/alanine rich; PAQ, proline/alanine/glutamine rich

The translational regulation of mRNAs containing CPE sequences in their 3′UTRs was first described in studies using oocytes from the African clawed frog *Xenopus laevis* (Fig. 1 A). In addition to the CPEB proteins, a number of other factors were found to bind to the 3′UTRs of CPE-containing mRNAs and to play important roles in regulation. These include poly(A) polymerase (PAP), poly(A) ribonuclease (PARN), the scaffold protein Symplekin, the translational repressor Maskin, and the poly(A)-binding protein (PABP). When PARN activity prevails over PAP activity in an RNP complex, the poly(A) tail is shortened and the amount of bound PABP decreases [1]. Maskin binds to the translation initiation factor eIF4E, preventing the assembly of the translation initiation complex [2], and interacts with the CPEB protein and PABP. Conversion of a translationally silent RNP complex into a complex that promotes translation requires an activation signal. The CPEB proteins are activated by phosphorylation, and this event leads to changes in the composition of the RNP complex. PARN dissociates from the complex, and PAP activity becomes predominant, leading to elongation of the poly(A) tail and a consequent increase in the amount of PABP bound to mRNA. As a result, affinity of Maskin for eIF4E decreases, thus allowing activation of translation. There are variations to this general scheme across different organisms and cell types. For example, neuroguidin (Ngd) is included in the RNP complex instead of the Maskin protein in *Drosophila* neurons [3]. Likewise, protein kinases involved in phosphorylation of the CPEB proteins differ between species.

Based on phylogenetic analysis, the CPEB proteins can be divided into two subfamilies [4, 5]. The CPEB1 subfamily includes the *Drosophila* Orb protein and the CPEB1 proteins of mammals, including humans. These proteins are mainly involved in regulation of translation during oogenesis and embryonic development [6]. In addition, they recently were found in neuronal synapses and shown to participate in the development and function of the nervous system [7]. The proteins Orb2 of *Drosophila* and CPEB2–4 of mammals belong to the CPEB2 subfamily. CPEB2 and CPEB4 also regulate mRNA translation, although the mechanisms of regulation are different [8]. CPEB3 is expressed in the brain and localized in postsynaptic densities; the protein plays a crucial role in learning and memory formation. The *Drosophila* Orb2 protein is involved in learning and long-term memory and is believed to be a component or regulator of synaptic tagging [4]. The similarities in the functioning of the CPEB proteins between phylogenetically distant species suggest that key aspects of memory formation may be conserved throughout evolution. Here, we consider the functions of the CPEB proteins in *Drosophila* and mammals and review the role of the CPEB proteins in pathological processes of the human nervous system and potential treatment approaches for these disorders.

## Biochemical properties of CPEB proteins

### CPEBs are RNA-binding proteins

According to different estimates, 20–40% of all *Xenopus*, mammalian, and human mRNAs are subject to CPE-mediated translational control [9]. In experiments on *Drosophila* cell culture, Orb bound to 2693 transcripts with CPE and Orb2 bound to 1639 target mRNAs with CPE sequences [10]. In vertebrates, the consensus sequence for binding with CPEB1 is U_4-5_A_1-3_U [11, 12]. Weak binding to CPEB1 was also shown for non-consensus CPEs, such as UUUUACU, UUUUAACA and UUUUAAGU [9, 13]. CPEB1 and CPEB4 recognize the same CPEs, but have different affinities for them [14, 15]. CPEB3 and CPEB4 can recognize and bind to mRNA secondary structures [8]. Analysis of the interactions between mRNAs and the CPEB proteins of *Drosophila* has shown that Orb and Orb2 bind to different CPE motifs [10]. For Orb, the sequence conforms to UUUUA_1-3_U, overlapping with the consensus CPE of vertebrates, while Orb2 binds to the canonical UUUUAAAU sequence and the noncanonical UUUUGU [10]. Thus, multiple target transcripts for the CPEB1 and CPEB2–4 subfamilies of the CPEB proteins overlap each other, but are not identical.

The position of CPEs in the 3′UTR of mRNA influences the efficiency of CPEB function. In vertebrates, a polyadenylation site (PAS) is located at the end of the 3′UTR. PAS consists of several *cis*-elements, including an AAUAAA hexamer (rarely, AU/GUAAA or UAUAAA), a U/GU-rich downstream sequence element, and a cleavage site positioned at a distance of 15–30 nt from the hexamer [16] (Fig. 1b). The distance between CPE and the hexamer determines the efficiency of polyadenylation and translation, while the distance between two CPEs determines the efficiency of translational repression [9]. The optimal distance between two CPE motifs for repressing translation is 10–12 nt. The optimal distance from the CPE to the hexamer for activating polyadenylation is 6–25 nt, while a distance of more than 120 nt was found to be nonfunctional [5, 9] (Fig. 1 B). The distance between the CPE motif and the hexamer appears to be determined by interactions between CPEB and the cleavage and polyadenylation specificity factor (CPSF), which binds to the hexamer [17]. Proteins of the CPSF complex were identified in *Drosophila* [18], but their interactions with Orb and Orb2 were not reported. Therefore, the effective distance between CPE and the hexamer for translational activation may differ from that described above for vertebrates.

### CPEBs are prion-like proteins

All CPEB family proteins have two RRM-type RNA-binding domains and a zinc finger domain at the C-terminus, but differ, often substantially, in their N-terminal sequence. While the former is highly conserved, this is not true for the N-terminus. Some members of the CPEB family are prion-like proteins due to the presence of polyglutamine- or polyalanine-rich domains at the N-terminus. Prions are capable of forming stable conformations with different functions. Importantly, prions have the ability to induce conformational transformations in normal versions of themselves, converting to a prion form [19]. A protein in a prion conformation, even in minimal amounts, can act as an oligomerization center, thereby maintaining a pool of proteins in the prion conformation. Prion aggregates are characterized by a high stability and high resistance to chemical agents and intracellular proteases, these properties allow prions to remain in cells for a long time. A prion-like domain is found in the N-terminal sequence of *Drosophila* Orb2 and the mouse and human CPEB3 proteins (Fig. 1c). These proteins differ in biophysical properties, and we will therefore consider each of them individually.

The Orb2 protein has two isoforms, Orb2A and Orb2B (Fig. 1c). Both isoforms have the prion-like polyglutamine domain at the N-terminus. However, the isoforms differ in biophysical properties: Orb2A forms amyloids more efficiently than Orb2B both in vitro and in vivo [20]. A sequence of 8 amino acids preceding the polyglutamine domain at the N-terminus of Orb2A is required for triggering protein oligomerization, and the polyglutamine domain serves as a substrate in this process, with both protein isoforms forming Orb2A–Orb2B complexes in neurons [20, 21]. Incubation of Orb2 monomers with trace amounts of Orb2 fibrils led to aggregation of the monomers; however, monomers did not aggregate without addition of fibrils. Orb2 fibrils did not induce aggregation of the human prion-like protein RBM3 [22], indicating a high specificity of the reaction and low toxicity of the resulting aggregates. The formation of protein aggregates in the brain is often associated with various neurodegenerative disorders (Huntington's disease, Alzheimer's disease, Parkinson's disease, and prion diseases). Studies of the amyloid structure formed by Orb2 have shown that it differs from beta-amyloids observed in Alzheimer's disease: fibrils of the latter have a hydrophobic core, while the core of Orb2 fibrils is hydrophilic, and a decrease in pH leads to destabilization of Orb2 fibrils or even their breakdown [22].

Similar to Orb2, mouse CPEB3 forms fibrils [23]. The prion-like domain of CPEB3 consists of three functional parts. Two parts of the domain are required for the formation of amyloids, and one of them also induces the aggregation of monomers into oligomers. A region between these two parts is required for the localization of CPEB3 in cells [23]. The mouse and human CPEB3 proteins are homologs with 89% sequence identity. In human CPEB3, polyalanine and polyglutamine tracts are required for the induction of aggregation [24]. A high frequency of proline in the mammalian CPEB3 prion domain restricts amyloidogenesis in aggregation-prone regions of the protein to prevent excessive oligomerization, which can damage neurons. Phosphorylated serine residues might be necessary for controlling the transition from the monomeric to an oligomeric form. In contrast to Orb2, functional amyloids formed by human CPEB3 do not differ from pathological amyloids, as shown by in vitro experiments [24]. Both functional and pathological amyloids form toxic metastable oligomer species. However, toxic intermediates of functional amyloids are extremely transient, while toxic conformers of pathological amyloids are long-lived, lasting weeks. Metastable CPEB3 toxic oligomers were long-lived, similar to pathological amyloids [24]. The cytotoxic effect of CPEB3 oligomers was confirmed in a neuroblastoma cell culture. Addition of CPEB3 amyloids to the cell medium increased the number of necrotic cells. A complex molecular apparatus, some components of which are still unknown, allows cells to avoid the cytotoxic effect of functional amyloids [25].

## Functions of CPEB proteins in brain

### CPEB proteins in normal development of nervous system

**Fig. 2:**
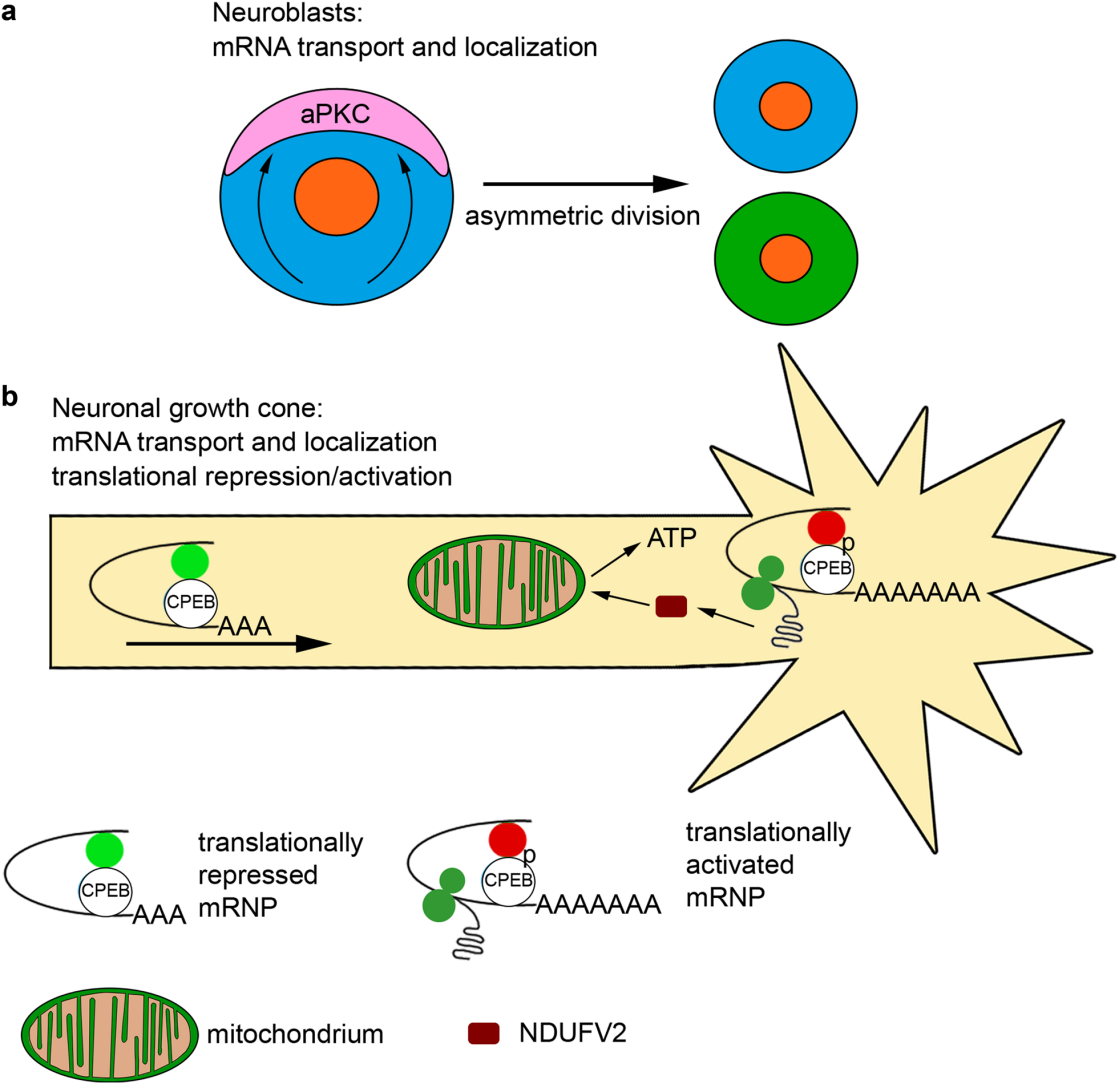
Functions of the CPEB proteins during development of the nervous system. **a** Orb2 participates in neuroblast asymmetric division, transporting and localizing the polarity complex protein aPKC. **b** The CPEB proteins transport and localize their target mRNAs to neuronal growth cones in both vertebrates and invertebrates. CPEBs inhibit or promote protein synthesis necessary for neuronal growth and branching. CPEB1 regulates expression of the NDUFV2 gene for a subunit of mitochondrial complex I involved in ATP synthesis

The CPEB proteins are primarily involved in neurogenesis of various organisms in the monomeric forms: these are Orb2B monomers for *Drosophila* [21, 26] and CPEB1 which lacks a prion-like domain, for mammals. One of the functions of CPEB in the monomeric form is to participate in the transport and/or anchoring on site of localized mRNAs (Fig. 2a, b). The CPEB proteins perform these functions as part of larger RNP complexes. A recent review [27] summarizes what is known about the composition of the RNP complexes in dendrites, which are associated with and/or regulated by the CPEB1 protein. CPEB1 interacts with 11 transcripts involved in long-term potentiation, memory formation, and synapse morphogenesis. The CPEB proteins are transported as part of RNP complexes from the nucleus to the cytoplasm [27]. Experiments on rat hippocampal neuron cultures have shown that CPEB1 binds to β-catenin mRNAs, thus leading to their localization to the growth cones of neurons [28]. β-Catenin is required for neuronal growth and branching.

The participation of CPEBs in mRNA localization appears to be important for asymmetric division of neuroblasts in *Drosophila* (Fig. 2a). A key role in asymmetric division is played by polarity complex proteins Par/aPKC/Baz (localized in the apical part of the cell) and Numb/Pros/Brat (basal localization), which determine the spindle orientation prior to cell division [29]. The *Drosophila* CPEB proteins Orb and Orb2 interact with mRNAs that encode many of the factors involved in asymmetric cell division [10]. Asymmetric division of neuroblasts is disrupted in embryos of *orb2* mutant flies [30]. One of the likely Orb2 targets in this process is the mRNA for PAR protein atypical protein kinase C (aPKC). In *orb2* mutants, aPKC does not concentrate at one of the neuroblast poles, and the spindle is oriented arbitrarily, resulting in disruption of asymmetric cell division. It is assumed that *orb2* can promote the accumulation of aPKC at the apical pole during asymmetric division of neuroblasts [30]. Another transcript target of Orb2 is the Brat polarity factor. An *orb2* deletion leads to disturbances in the formation of neuromuscular connections and synapses in the central nervous system, thought to be due to Brat mislocalization [31].

In addition to controlling the mRNA localization, the CPEB proteins are involved in regulating translation of mRNA localized in neurons (Fig. 2b). The CPEB proteins inhibit mRNA translation in their basal state by interacting with the deadenylation complex, which shortens the poly(A) tails of mRNAs [26, 32]. When phosphorylated by various kinases, the CPEB proteins function as translational activators. It was shown that the CPEB1 protein is not only involved in the localization of the β-catenin mRNA in the growth cones of neurons, but it also increases the level of its translation [28]. When CPEB1 expression is disturbed, branching of neurons stops [28]. In *Drosophila,* Orb2 negatively regulates Brat expression, and the level of Brat expression increases in *orb2* mutants. It was assumed that Orb2A–Orb2B oligomers are involved in regulation of *brat* expression [31]. CPEB1 was found to regulate polyadenylation and translation of the NDUFV2 mRNA, which encodes a subunit of mitochondrial complex I involved in ATP synthesis [32]. The level of ATP in the brain is significantly reduced in CPEB1 knockout mice compared to wild-type mice, but no changes occur in the muscles and liver. The branching and growth of dendrites is impaired, and the changes observed in the mutant mice are similar to those described in the aforementioned studies [32]. Other studies indicate that CPEB1 is involved in translational regulation of the DSCAM mRNA, which encodes a cell adhesion molecule involved in dendritic branching [33]. The gene coding for this protein is one of the candidate genes for the pathogenesis of Down syndrome.

### Place of CPEBs in molecular network behind memory

Short-term and long-term memory are distinguished based on the duration of information storage. At the molecular level, the distinction is that activation or inhibition of gene expression is required for long-term memory, but not for short-term memory [34]. Synaptic plasticity is the main mechanism that underlies the phenomenon of memory and learning [35]. Synaptic plasticity is the ability to alter the strength or efficiency of signal transmission at synapses. In invertebrates, two forms of synaptic plasticity were conventionally distinguished: short-term and long-term synaptic facilitation associated with the formation of short-term and long-term memory, respectively. In mammals, plasticity can be classified with respect not only to its duration, but also to its form: potentiation (increased synaptic transmission) or depression (decreased transmission efficiency). Recent studies provided a deeper insight into synaptic plasticity in invertebrates, suggesting that it can also be classified in terms of potentiation and depression [35]. Key insights into memory formation in invertebrates came from studies on the sea slug *Aplysia* and *Drosophila*. The advantage of *Aplysia* for researchers is that its nervous system has a quite simple organization. On the other hand, key players in the formation of long-term memory in the *Aplysia* nervous system—such as cAMP, protein kinase A (PKA), CREB1, CREB2, MAPK (Mek1/2 in mice), and CPEB—perform similar functions in other animals, such as *Drosophila* and mice [34].

In *Aplysia*, serotonin is released in response to stimulation of the presynaptic terminal of a sensory neuron (from an intercalary neuron) and induces the synthesis of cAMP from ATP [34]. cAMP activates PKA, which phosphorylates proteins in the presynaptic terminal, resulting in short-term facilitation of synaptic transmission [36]. Thus, one stimulus leads to local short-term changes near the synaptic gap. Five applications of serotonin to a sensory neuron at 10-min intervals result in long-term facilitation of synaptic transmission lasting for more than 24 h [37]. Repetitive stimuli lead to changes in the neuron nucleus, such as induction of the transcriptional activator CREB1 (cAMP response element binding protein 1) and inactivation of the transcriptional repressor CREB2 [34], triggering the synthesis of mRNAs necessary for long-term facilitation (for more detail on the participants in the process, see [34]). The newly synthesized mRNAs are transported from the neuron body to the synapses; the specificity of this process may differ for different mRNAs [38]. Using a bifurcated sensory neuron forming synapses with two motor neurons, an individual synapse, rather than the entire neuron, was identified as a unit of memory storage [37]. The synthesis of proteins from the transported mRNAs occurs only at an activated synapse. The finding that long-term facilitation takes place in individual synapses led to a synaptic tagging hypothesis, which assumes that synapses involved in long-term memory undergo molecular and structural changes [37, 39]. Protein synthesis near a tagged synapse extends over a period of days, facilitating the growth of the synapse and the formation of new synaptic contacts [40].

One potential problem with the mechanism that involves protein synthesis in synapses as a basis for maintaining long-term memory is how this memory could be stored for days or years if protein decay would occur within a shorter period of time. In 1998, Peter Tompa and Peter Friedrich proposed a prion theory of memory [41]. The theory assumes that the prions that are involved in memory adopt a non-toxic conformation, in which they can renew themselves indefinitely in synapses, catalyzing the conformational transformation of newly synthesized proteins into prion proteins. The discovery of the prion-like structure of the CPEB proteins provided evidence potentially consistent with this theory. It is considered that the CPEB proteins, first, are required for the mRNA transport within RNP complexes and the regulation of mRNA translation and, secondly, are a key component or regulator of synaptic tagging.

### Molecular mechanism of CPEB participation in memory formation

**Fig. 3:**
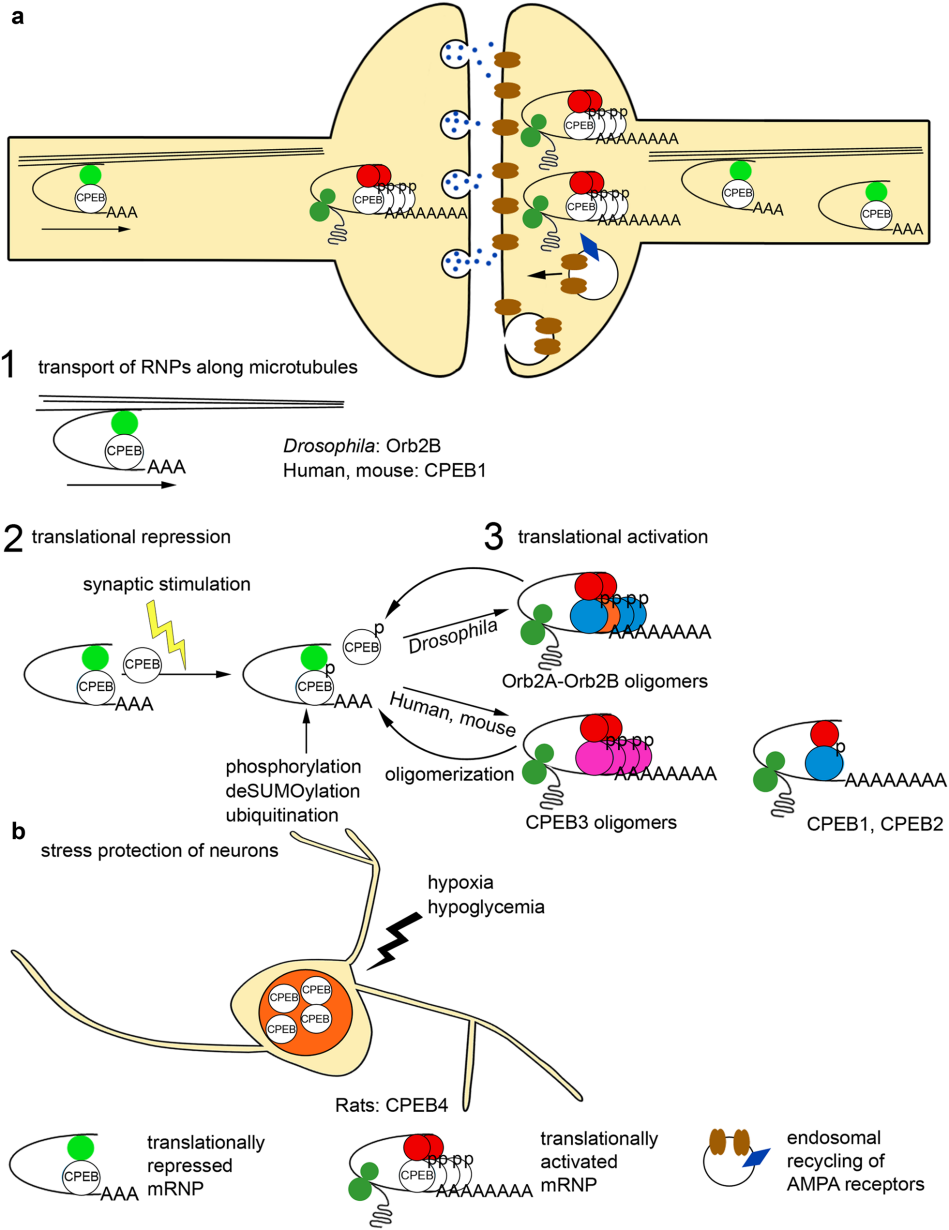
Functions of the CPEB proteins in neurons. **a** Scheme of a synapse summarizes the processes described below. 1. CPEB transports translationally silent mRNA as part of a RNP complex along microtubules with the help of the kinesin and dynein motor proteins. 2. CPEBs act as translational repressors in their basal state and are activated by different enzymes after synaptic stimulation. *Drosophila* Orb2 is phosphorylated by protein kinase LimK; vertebrate CPEBs are phosphorylated by kinase Aurora A, ubiquitinated by Neuralized1, and deSUMOylated by proteases. 3. Activated CPEBs induce polyadenylation of mRNA and activation of protein synthesis. Orb2 and CPEB3 form oligomers, while CPEB1 and CPEB2 function as monomers. Endocytic vesicles with receptors on the scheme of a synapse demonstrate the role of CPEB2 in regulation of the GRASP1 protein, involved in recycling and maintaining the surface level of AMPA receptors. **b** CPEB4 is necessary for neuronal survival under stress conditions of hypoxia and hypoglycemia and is localized in the nuclei of neurons

Both neurogenesis and the normal functioning of the brain require the transport and localization of mRNAs from the cell body of neurons to dendrites and axons (Fig. 3a). The involvement of CPEB1 in the mRNA transport through the microtubule system to dendrites of nerve cells was shown in a mouse cell culture, where RNP complexes interacted with the motor proteins dynein and kinesin [42]. As part of RNP complexes, CPEB1 is involved in transport of mRNA of the brain-derived neurotrophic factor (BDNF), which is required for long-term memory and is one of the components of synapse tagging [43–45]. The level of the Orb2B isoform, which is involved in mRNA transport, in the *Drosophila* brain is 100 times higher than the concentration of Orb2A required for a local regulation of translation [20, 46].

The mRNAs that are transported and localized by CPEB-containing RNP complexes are in a translationally dormant state. For example, mouse mRNAs are localized in P-bodies, which contain monomeric CPEB3 [47]. CPEB3 is a negative regulator of several plasticity-related proteins, including NR1, NR2A, NR2B, and PSD95 [48]. NR1, NR2A and NR2B are thought to form di- or heterotrimeric NMDA receptors, while PSD95 is a scaffolding protein that functions in the clustering of receptors, ion channels, and associated signaling proteins. Local protein synthesis in synapses is activated by phosphorylation of the CPEB proteins in response to synaptic stimulation (Fig. 3a). In hippocampal neurons, CPEB phosphorylation by protein kinase Aurora A was observed in response to stimulation of NMDA receptors, resulting in activation of local translation of CAMKIIα mRNA [49]. As found by McEvoy et al. [50], CPEB1 point mutations that disrupt phosphorylation by Aurora A protein kinase in Purkinje cells of the mouse cerebellum impair the long-term depression phase and result in changes in the morphology of cerebellar neurons. Mice with the mutant CPEB1 protein expressed in Purkinje cells show disorders in spatial coordination and motor learning [50]. In *Drosophila*, Orb2A is phosphorylated and stabilized during synaptic activation by protein kinase LimK, which thus causes a local increase in Orb2A concentration near synapses [51]. An increase in Orb2A concentration triggers Orb2A–Orb2B oligomerization. Orb2A–Orb2B amyloids bind to the 3′UTRs of target mRNAs, preventing deadenylation of transcripts. Oligomers interact with the CG4612 protein, which promotes mRNA polyadenylation and activation of translation of many Orb2 target transcripts [26].

Like Orb2A, CPEB3 forms aggregates in response to neuronal stimulation. CPEB3 interaction with the actin cytoskeleton is required for prion formation, while CPEB3 oligomers activate translation of actin mRNA [23]. The results obtained by Fioriti et al. [52] confirmed the formation of CPEB3 amyloids in response to neuronal stimulation in mouse hippocampal neurons. The ability of CPEB3 to form aggregates depends on the extent of its SUMOylation. When highly SUMOylated, CPEB3 is a monomer which localizes in P-bodies where helps to repress translation [47]. When neurons are stimulated, the level of SUMOylation decreases and CPEB3 aggregates, leading to translational activation of target mRNAs [53]. The activation of CPEB3 by ubiquitin ligase Neuralized1 (Neurl1) was observed in the brain of mice [54]. A *Drosophila* ortholog of Neurl1 (Neuralized) is also required for the formation of long-term memory [55]. The activation of CPEB3 triggers the synthesis of the GluA1 and GluA2 subunits of AMPA receptors and the formation of new synaptic spines [52, 54]. The AMPA receptors are a subtype of ionotropic glutamate receptors, which are abundant in the postsynaptic membrane on dendritic spines and play a critical role in long-term potentiation [56]. Conditional CPEB3 knockout mice (with no CPEB3 mRNA expression in the hippocampus and cortex) show a significant impairment in the formation of long-term memory and long-term potentiation [52]. Moreover, CPEB3 was shown to play a role in the formation of not only long-term memory, but also of short-term memory in humans [57]. Since amyloids are stable and capable of self-maintenance, synapse-specific and self-sustaining activation of mRNA translation of CPEB protein target genes ensures the accumulation of proteins near the synapse, which is necessary for long-term potentiation associated with learning and long-term memory.

The most detailed experiments to confirm the role of the CPEB proteins in long-term memory were carried out on *Drosophila*. Li et al. [58] found that an insertion of a transgenic construct containing a gene that facilitated Orb2 aggregation in *Drosophila* resulted in a significant increase in the rate of acquisition of long-term memory. Evidence that the maintenance of memory also requires Orb2 comes from studies on a fly strain carrying an *orb2* gene that contains recognition sequences for tobacco mosaic virus protease [58]. When the protease was activated in flies a day after the initial formation of long-term memory, a significant decrease in memory was observed. This finding suggests that Orb2 is required for the maintenance of memory after its consolidation. The loss of already formed memories could be associated either with the inability to recall them again or with the memory being “damaged”. If Orb2 is only needed to recall memories, then it should be possible to inactivate Orb2 during memory formation and then turn on *de novo* Orb2 expression when the memory is recalled. However, activation of the protease before or during training, with its subsequent inactivation, was found to disrupt memory formation. This suggests that Orb2 is also necessary for the formation of memory [58].

The CPEB proteins were also found to regulate expression and the functioning of AMPA receptors. The CPEB2 protein activates translation of mRNAs encoding the endosomal protein GRASP1, implicated in recycling and maintaining the surface level of the AMPA receptors [59]. The number of the GluA1 and GluA2 subunits of AMPA receptors is decreased on the surface of neurons in forebrain-restricted conditional CPEB2 knockout mice. This decrease correlates with impairments in hippocampus-dependent synaptic plasticity and spatial memory [59]. Regulation of the GluA2 mRNA by CPEB3 results in a gradient distribution of the AMPA receptors in dendrites, providing a difference in the conductance of action potential depending on the dendrite length [60]. In distal dendrites, the concentration of GluA2 is high near the postsynaptic membrane and decreases towards the neuronal soma [60]. In addition to their involvement in the formation of memory, the CPEB proteins exhibit neuroprotective properties under stressful conditions, while the exact mechanism is unclear (Fig. 3b). CPEB4 is expressed at a high level in neurons of the brain and spinal cord of mice, although CPEB4 knockout mice develop normally and do not have memory impairments [61]. Under stress caused by hypoxia and hypoglycemia (ischemia), CPEB4 concentrates in the nuclei and is required for the survival of nerve cells [62].

### CPEB proteins and pathologies of the central nervous system

As indicated in Table 1, several diseases of the nervous system are associated with the malfunctioning of the CPEB proteins, and these will be considered below.

**Table 1:** Nervous system disorders in which the CPEB proteins may be used as targets for gene therapy

Disease	CPEB protein	CPEB expression in disease	Participation of CPEB in the pathogenesis or correction of the disease	References
Autism spectrum disorder	CPEB4	Decreased level of the protein, but not of the mRNA	Mis-splicing of CPEB4 mRNA leads to deregulated expression of CPEB4 target genes involved in ASD	[63]
Gliomas	CPEB1	Decreased expression	Increased CPEB1 expression reduces proliferation and self-renewal of tumor cells	[64, 65]
	CPEB4	Increased expression	Decreased CPEB4 expression leads to reduction in tumor size, cell proliferation rate, microvessel density, and tumor cell invasion and migration	[66–68]
Fragile X syndrome	CPEB1	Expression is normal	Deletion of the CPEB1 gene in mice suppresses pathological processes associated with the syndrome	[73, 74]
Huntington's disease	CPEB1–4	Decreased level of the protein in the cytoplasm	Increased expression of CPEB proteins reduces the pathogenicity of protein aggregates in *Drosophila*	[80]

Autism spectrum disorder (ASD) is a polygenic disease in which variants of hundreds of genes are involved, each making a minimal individual contribution to the development of the disease. ASD is a developmental disability that manifests itself in childhood and is characterized by a persistent deficit in the ability to initiate and maintain social interaction, as well as limited interests and often repetitive behaviors. A recent study on humans and mice showed that the disorder correlates with impaired CPEB4 mRNA splicing [63]. The amount of the CPEB4 protein in brain cells is decreased in people with ASD, even though the levels of its mRNA are increased. Analysis of the CPEB4 mRNAs in ASD individuals showed that the relative abundance of transcripts with a splicing variant that includes the 4th microexon is decreased. This microexon is 24 nucleotides in length and is incorporated into the neuronal CPEB4 mRNAs. CPEB4 target mRNAs have shortened poly(A) tails and reduced levels of translation. Findings of experiments on mice confirm that impaired translation of CPEB4 target mRNAs is associated with a disturbance in the ratio of CPEB4 splicing variants (an increase in the level of transcripts without the 4th microexon); when this ratio remains undisturbed, a general decrease in CPEB4 expression does not lead to a decrease in the expression level of CPEB4 target mRNAs. Analysis of CPEB4 target genes revealed a large number of candidate genes involved in the development of ASD. Thus, while ASD is considered a polygenic disorder, CPEB4 mis-splicing leads to misregulation of multiple target genes, many of which are ASD-associated candidate genes [63].

The CPEB family proteins play an important role in cell proliferation and differentiation; therefore, they are involved in the development of tumors, which makes them potential targets for gene therapy. Expression of the CPEB1 and CPEB4 genes influences the formation of gliomas in the brain. Glioblastoma multiforme, the most common and aggressive brain tumor, has a significantly reduced level of CPEB1 gene expression as compared with normal brain cells [64, 65]. Induction of CPEB1 expression upon transfection of a glioblastoma cell culture with a plasmid results in a two-fold reduction in the rate of cell proliferation. One plausible explanation for the reduction in proliferation is that CPEB1 binds to the 3′UTR of the mRNAs for the tumor suppressor gene cyclin-dependent kinase inhibitor p27^Kip1^, activating its translation. CPEB1 competes for binding sites in the p27^Kip1^ 3′UTR with the oncogenic miR-221 and miR-222 microRNAs which repress translation of the p27^Kip^ mRNAs [64]. The difficulty in treating glioblastoma multiforme is associated with the inability of available methods to remove glioblastoma stem cells (GSCs), which are capable of self-renewal and give rise to various differentiated cell lines that form the tumor. An induced increase in CPEB1 expression in GSC culture suppresses the ability of GSCs to self-renew, leading to their differentiation [65].

Unlike with CPEB1, expression of CPEB4 is increased in gliomas [66–68]. A decrease in CPEB4 expression in a glioma cell culture led to reductions in tumor size, cell proliferation rate, and microvessel density [67]. In experiments on glioblastoma and astrocytoma cell cultures, inhibition of CPEB4 expression by siRNAs decreased invasion and migration of tumor cells [66, 68]. Increased expression of CPEB4 in cancer cells can be used for a specific activation of oncolytic viruses in these cells [69]. The immediate-early genes are the first viral genes transcribed after invasion of a virus into the cell. In an oncolytic adenovirus, the 3′UTR of the wild-type E1A early response gene (AdWT virus) was replaced by the *Xenopus* cyclin B1 3′UTR (AdCPE virus), which contains two consensus CPEs and one nonconsensus CPE. This combination of CPEs provides translational repression of the transcript by unphosphorylated CPEB1 and activation upon binding with CPEB4. Intravenous injection of the AdCPE virus in mice resulted in a significant reduction in tumor growth and was less toxic than injection of the AdWT virus. The levels of the pre-mRNA of the AdWT and AdCPE viruses were the same in healthy animal cells, while the mature mRNA level was reduced in cells infected with the AdCPE virus, suggesting a destabilizing effect of CPEB1 on the E1A mRNA [69].

A current trend is to study the CPEB proteins for potential use as a target for gene therapy. Fragile X syndrome (Martin–Bell syndrome) is an inherited genetic disorder that manifests itself in intellectual disability and is associated with a mutation in one gene located on the X chromosome [70]. Inactivation of the *FMR1* (fragile X mental retardation-1) gene underlies fragile X syndrome and results from an increase in the number of CGG repeats in the 5'UTR of the gene [71]. The RNA-binding protein FMRP encoded by the *FMR1* gene is a translational repressor. The inactivation of *FMR1* is accompanied by a 20% increase in protein synthesis in the hippocampus [72]. When modeling fragile X syndrome in mice (*FMR1* knockout), mutations of the *CPEB1* gene was found to reduce the pathological processes associated with the syndrome [73]. Recent studies showed that the “rescue” of double knockout mice (*FMR1* and *CPEB1*) occurs at the level of RNA stabilization. In the cerebral cortex of *FMR1* knockout mice, the synthesis and maturation of mRNA remain at the same level, while an increase in the rate of mRNA degradation is observed for about 700 genes [74]. A CPEB1 deletion restores the balance of mRNA translation, but the mechanism of this phenomenon is not completely clear. In this respect, it is interesting to note that a decrease in CPEB expression can be achieved by manipulating the levels of miRNAs, and this could be a promising approach to gene therapy [75]. Studies on human cell cultures showed that miR-92 and miR-26 bind to the 3'UTRs of the CPEB2, CPEB3, and CPEB4 mRNAs decreasing expression of these CPEB proteins [76]. miR-22 represses CPEB expression in synapses of *Aplysia* while the synapses are inactive, thereby participating in the maintenance of synaptic plasticity [77].

Huntington's disease is a chronic neurodegenerative disease associated with progressive death of brain cells [78]. The cause of the disease is an increase in the number of glutamine (Q)-encoding CAG repeats in an exon of *huntingtin* (*htt*). In a pathological process, the number of glutamine coding triplets in *htt* varies from 36 to 180 [79]. The polyglutamine-containing protein Htt forms aggregates, leading to an imbalance in protein synthesis and decay in cells and subsequent death of neurons. A recent study on a *Drosophila* cell culture revealed absorption of the Orb2A protein on the surface of HttQ138 aggregates [80]. Upon expression of the human CPEB1–4 proteins in the same cell culture, the CPEB proteins colocalized with HttQ138 aggregates. It was assumed that the colocalization of the CPEB proteins with HttQ138 aggregates led to a decrease in the concentration of the CPEB proteins in the cytoplasm, which in turn resulted in a disruption in the regulation of their target mRNAs. Increased expression of Orb2A and Orb2B led to a significant decrease in the *Drosophila* mortality rate at the pupal stage in the presence of HttQ138, increasing the number of hatched flies from 4.8% to 60 and 51% (for increased expression of Orb2A and Orb2B, respectively). The amount of HttQ138 aggregates did not decrease, but the balance of protein synthesis in cells was restored [80].

## Conclusions

The functioning of the CPEB family proteins is essential at all stages of ontogeny. CPEBs play an important role in the formation and maintenance of cell polarity, participating in mRNA transport and localization, translational repression or activation of target mRNAs [30, 81–83]. In the nervous system, this function is manifested in the participation of the CPEB proteins in neurogenesis and the functioning of neurons. Much attention is devoted now to the role that the prion-like conformation of these proteins plays in the formation of long-term memory.

The CPEB proteins participate in the translational control of a wide range of mRNAs and, therefore, are involved in pathologies of the nervous system. Moreover, disturbances in the functioning of the CPEB proteins cause other pathological processes, including carcinogenesis, tumor invasion, and angiogenesis. In the case of rectal cancer, breast cancer, and gliomas, the expression levels of several CPEB proteins change simultaneously, which is indicative of interactions between them in the oncological process [67, 84–86]. The role of the CPEB proteins in certain liver diseases and metabolic disorders (e.g., hepatosteatosis) was also revealed [87].

Thus, investigation of the role of the CPEB proteins is an extremely important fundamental task that opens up prospects for understanding the molecular mechanisms of the formation and functioning of the nervous system and other body systems, as well as for finding ways to treat a wide range of diseases.

## Data Availability

Not applicable.

## Abbreviations

UTR: Untranslated region
RRM: RNA-recognition motif
ASD: Autism spectrum disorder
RNP: Ribonucleoprotein
CPE: Cytoplasmic polyadenylation element
CPEB: Cytoplasmic polyadenylation element binding
CPSF: Cleavage and polyadenylation specificity factor
PABP: Poly(A)-binding protein
aPKC: Atypical protein kinase C
GSCs: Glioblastoma stem cells
